# Generation Scotland: Linking all the records we can

**DOI:** 10.23889/ijpds.v8i2.2347

**Published:** 2023-09-14

**Authors:** Archie Campbell, Robin Flaig, Cathie Sudlow

**Affiliations:** 1 Ulster University, Belfast, United Kingdom; 2 St George's University, London, United Kingdom; 3 Hospital Lillebaelt, Odense, Denmark

## Objectives

We started a family-based genetic epidemiology study in 2006-11 which recruited ~24,000 adult volunteers from ~7000 families across Scotland with consent for follow-up through medical record linkage and re-contact. In 2022-23 we are recruiting another 20,000, with consent extended to administrative records, with age range now 12+.

## Methods

Original volunteers completed a demographic, health and lifestyle questionnaire, provided biological samples, and underwent detailed clinical assessment. The samples, phenotype and genotype data form a resource for research on the genetics of conditions of public health importance. This has become a longitudinal dataset by linkage to routine NHS hospital, maternity, lab test, prescriptions, dentistry, mortality, imaging, cancer screening, GP data records, Covid-19 testing and vaccinations, as well as follow-up questionnaires. The new wave of recruitment is all online and can be done on a smartphone, with DNA from saliva collected by post. Teenagers aged 12-15 can join with parental consent.

## Results

GWAS has been done on quantitative traits and biomarkers, with DNA methylation data and proteomics available for most of the cohort. Our “CovidLife” surveys collected data on effects of the pandemic.

Researchers can find prevalent and incident disease cases and controls, to test research hypotheses on a stratified population. They can also do targeted recruitment of participants to new studies, including recall by genotype. We have established and validated E-HR linkage with the NHS Scotland CHI Register,,overcoming technical and governance issues in the process. We contribute to major international consortia, with collaborators from institutions worldwide, both academic and commercial. Recruits are asked to give consent to linkage to other administrative data, and reuse of samples from routine NHS tests for medical research.

## Conclusion

We plan to extend the linkage process to include other administrative data from national datasets as and when approvals are obtained. New types of data can also be collected by online questionnaires. The Research Tissue Bank resources are available to academic and commercial researchers through a managed access process.

